# Melanocortin-4 Receptor in Spotted Sea Bass, *Lateolabrax maculatus*: Cloning, Tissue Distribution, Physiology, and Pharmacology

**DOI:** 10.3389/fendo.2019.00705

**Published:** 2019-10-18

**Authors:** Kai-Qiang Zhang, Zhi-Shuai Hou, Hai-Shen Wen, Yun Li, Xin Qi, Wen-Juan Li, Ya-Xiong Tao

**Affiliations:** ^1^The Key Laboratory of Mariculture, Ministry of Education, Ocean University of China, Qingdao, China; ^2^Department of Anatomy, Physiology and Pharmacology, College of Veterinary Medicine, Auburn University, Auburn, AL, United States

**Keywords:** spotted sea bass, melanocortin-4 receptor, signaling, allosteric modulator, constitutive activity

## Abstract

Melanocortin-4 receptor (MC4R) plays important roles in regulation of multiple physiological processes including energy homeostasis, reproduction, sexual function, and other functions in mammals. Recent studies suggested that teleost MC4Rs have different physiological functions and pharmacological characteristics when compared to mammalian MC4Rs. In this study, we investigated spotted sea bass (*Lateolabrax maculatus*) MC4R (*Lm*MC4R) physiology and pharmacology. Spotted sea bass *mc4r* consisted of a 984 bp open reading frame encoding a protein of 327 amino acids. *Lm*MC4R was homologous to those of several teleost MC4Rs and human MC4R (hMC4R). qRT-PCR and *in situ* hybridization revealed that *mc4r* transcripts were highly expressed in the brain, followed by pituitary and liver. Brain *mc4r* transcripts were down-regulated in long-term and short-term fasting challenges. *Lm*MC4R was a functional receptor with lower maximal binding and higher basal activity than hMC4R. THIQ was not able to displace ^125^I-NDP-MSH but could affect intracellular cAMP accumulation, suggesting that it was an allosteric ligand for *Lm*MC4R. *In vitro* studies with spotted sea bass brain cells indicated that mRNA levels of *neuropeptide Y* and *Agouti-related peptide* were down-regulated by α-MSH. In summary, we cloned spotted sea bass MC4R, and showed that it had different pharmacological properties compared to hMC4R, and potentially different functions.

## Introduction

Melanocortin peptides are posttranslational products of proopiomelanocortin (POMC) that include α-, β-, and γ- melanocyte-stimulating hormones (α-, β-, and γ-MSH) and adrenocorticotropic hormone (ACTH) [reviewed in (1, 2)]. Melanocortin peptides exert their effects by activating melanocortin receptors (MCRs). Five MCRs have been cloned, named MC1R to MC5R based on the order in which they were first cloned [reviewed in (3, 4)]. MC4R belongs to Family A rhodopsin-like G protein-coupled receptors (GPCRs) and it primarily couples to the stimulatory G protein (G_s_) to activate adenylyl cyclase, leading to increased level of intracellular cyclic adenosine monophosphate (cAMP) to activate downstream protein kinase A (PKA). α-MSH, ACTH and other POMC-derived peptides are endogenous agonists and Agouti-related peptide (AgRP) is endogenous antagonist of MC4R. In addition, analogs of α-MSH and some small molecules have also been identified as MC4R ligands. [Nle^4^, D-Phe^7^]-α-MSH (NDP-MSH) is a superpotent analog of α-MSH that is widely used in pharmacological studies of MCRs (5). THIQ, (N-[(3R)-1,2,3,4-tetrahydroisoquinolinium-3-ylcarbonyl]-(1R)-1-(4-chlorobenzyl)- 2-[4-cyclohexyl-4-(1H-1,2,4-triazol-1-ylmethyl)piperidin-1-yl]-2-oxoethylamine), is a small molecule agonist (6).

Activation of neurons expressing neuropeptide-Y (NPY) and AgRP increases food intake, while activation of neurons expressing POMC decreases food intake in human and mice [reviewed in (7)]. POMC-derived peptides, such as α-MSH and ACTH, are anorexigenic by activating MC4R. In human, two groups independently reported that *MC4R* frameshift mutations are associated with severe early-onset obesity in 1998 (8, 9). Since then, a total of at least 175 distinct *MC4R* mutations have been identified from patients associated with obesity and other diseases [reviewed in (10, 11)]. Mice lacking *Mc4r* expression have increased food intake and decreased energy expenditure, resulting in obesity and hyperinsulinemia (12). In addition to regulation of energy balance, recent studies reported that MC4R is also involved in reproductive functions via regulating hypothalamus-pituitary-gonad axis and prolactin secretion (13–15).

MC4R and other MCRs have also been identified in tetrapods and teleosts. In tetrapods, all MCRs (MC1R-MC5R) have been identified and higher MC4R expression was observed in central nervous system (3, 16). In teleosts, MC4R is expressed in both central and peripheral tissues (17–23). In cavefish (*Astyanax mexicanus*), non-synonymous *mc4r* mutations cause increased appetite and starvation resistance (24). In zebrafish, overexpression of AgRP leads to obesity phenotype (25). Intracerebroventricular (i.c.v) injection of MC4R agonist decreases food intake, while injection of MC4R antagonist increases food intake in goldfish and rainbow trout (*Oncorhynchus mykiss*) (26, 27). These results suggest that teleost MC4R also acts as a regulator in energy balance. Teleost MC4Rs are also associated with the onset of puberty, growth and body size, and sexual behaviors in a species-specific manner in different teleosts (28, 29). We showed that administration of MC4R ligands to spotted scat can change expression of genes related to reproduction (30).

Our previous studies showed that teleost MC4Rs have different pharmacological characteristics from mammalian MC4Rs. For example, compared to human MC4R (hMC4R), teleost MC4Rs display high basal activities (20–23). Moreover, THIQ acts as an orthosteric agonist to activate mammalian MC4Rs; however, it activates teleost MC4Rs allosterically (20, 22). Therefore, in the present study, we used spotted sea bass, *Lateolabrax maculatus*, as an animal model to systematically investigate *Lm*MC4R physiology and pharmacology. We investigated mRNA expression and localization of *mc4r* in different tissues and changes in expression after fasting challenge. We also performed detailed pharmacological studies on *Lm*MC4R including ligand binding and signaling. We included hMC4R in these experiments for comparison. We also isolated brain cells where *mc4r* was expressed most abundantly and stimulated these cells with α-MSH to evaluate the transcriptional changes of several genes associated with growth and energy balance.

## Materials and Methods

### Gene Cloning and Sequence Alignment

All procedures involving fish followed the guidelines and were approved by the Animal Research and Ethics Committee of Ocean University of China (Permit Number: 20141201).

Total RNA was extracted from spotted sea bass brain using TRIzol (Invitrogen, Carlsbad, CA, USA). The concentration and integrity of total RNA were evaluated by the Agilent 2100 Bioanalyzer system (Agilent Technologies, Santa Clara, CA, USA). One microgram of RNA was used to synthesize first-strand cDNA using random primers and reverse transcriptase M-MLV with gDNA Eraser (TaKaRa, Japan). To amplify cDNA fragments of *mc4r*, PCR was performed and primers of *mc4r* were designed based on transcriptome databases (Table 1). PCRs were performed in a 25 μl mixture containing 1 μl cDNA, 0.5 μl of each primer, 2 μl dNTPs, 2.5 μl 10 × PCR buffer, 18.25 μl ddH_2_O, and 0.25 μl Taq DNA Polymerase (TaKaRa) with following program: initial denaturation at 94°C for 5 min, followed by 40 cycles at 94°C for 30 s, 60°C for 30 s and 72°C for 1 min. The reaction was terminated with a further extension of 5 min at 72°C. The amplification products were separated by 1.5% w/v agarose gel stained with ethidium bromide. Target fragment was purified by TIANgel Midi Purification kit (Tiangen, Beijing, China), and then subcloned into Trans1-T1 *Escherichia coli* (TransGen Biotech, Beijing, China). Two positive clones were sequenced on an ABI 3700 sequencer (Applied Biosystems, Foster City, CA, USA).

**Table 1:** The primers of *mc4r, agrp, npy*, and *18s* in spotted sea bass.

**Primer name**	**Primer sequence (5^′^-3^′^)**	**Application**
*mc4r*-f	CGGTGCTCATCTGCCTCATC	qRT-PCR
*mc4r*-r	CTTCATGTTGGCTCGCTGGT	qRT-PCR
*mc4r*-f	CGCATTTAGGTGACACTATAGA AGCGAAGACTTATCAGGCGAGGAC	ISH
*mc4r*-r	CCGTAATACGACTCACTATAGGG AGACAAGGAGGATGGTGAGGGTG	ISH
*agrp*-f	GATGGACACAGGCTCCTACGAC	qRT-PCR
*agrp*-r	GGCATTGAAGAAGCGGCA	qRT-PCR
*npy*-f	GAGGGATACCCGATGAAACCG	qRT-PCR
*npy*-r	CCTCTTTCCATACCTCTGTCTCG	qRT-PCR
*18s*-f	GGGTCCGAAGCGTTTACT	qRT-PCR
*18s*-r	TCACCTCTAGCGGCACAA	qRT-PCR

Multiple alignments of amino acid sequences of MC4Rs in different species were performed with DNAMAN 6.0 (Lynnon Biosoft, San Ramon, CA, USA). The percentage of similarity between amino acid sequences were calculated with DNAMAN 6.0. Phylogenetic tree based on amino acid sequences was constructed by Neighbor-joining and Maximum likelihood methods with Mega 6.0 software. The strength of branch relationships was assessed by bootstrap replication (N 1/4 1,000 replicates).

### Tissue Distribution of *mc4r*

Total RNA was extracted from fresh tissues (pituitary, brain, liver, kidney, spleen, intestine, muscle, gonads, gill, heart) and treated with RNase-free DNase I (Thermo Scientific Corp, Waltham, MA, USA). M-MLV Reverse Transcriptase (Promega, Madison, WI, USA) was used for cDNA synthesis with oligo-dT (12-18) primers. The cDNA was subsequently used for amplification using specific primers based on *mc4r* sequence from transcriptome database. *18s* mRNA expression was used as an internal reference for normalization (31). The quantitative reverse transcription PCR (qRT-PCR) reaction consisted of a total volume of 20 μl mixture containing 10 μl SYBR®FAST qPCR Master Mix (2X), 0.4 μl ROX reference dye, 2 ml template cDNA, 0.4 μl of each primer and 6.8 μl of nuclease-free water. PCR amplification was in a 96-well optical plate at 95°C for 5 s, followed by 40 cycles of 95°C for 5 s, 60°C for 30 s, and finally followed by a dissociation curve to verify the specificity of amplified products. qRT-PCR was performed using the StepOne Plus Real-Time PCR system (Applied Biosystems) and the 2^−ΔΔ*CT*^ method was used to analyze the relative expression (32).

### Localization of *mc4r* in Brain, Liver, and Pituitary

Brain, liver, and pituitary samples were fixed in buffered 4% paraformaldehyde for 24 h and then dehydrated with a graded series of ethanol solution (70–100%), cleared in xylene and embedded in paraffin. Seven-micron sections were cut for *in situ* hybridization. The primers for *in situ* hybridization of *mc4r* was listed in Table 1. Sense and antisense digoxigenin- (DIG)- labeled riboprobes were synthesized from the sequence (574–1,232 bp) of the *mc4r* using DIG RNA Labeling Kit (Roche Diagnostics, Mannheim, Germany). DIG *in situ* hybridization was performed as described previously (33). Briefly, the sections were rehydrated by a graded series of ethanol solution (100–70%) and then permeabilized with 0.1 M HCl for 8 min, followed by proteinase K (20 ng/μl) treatment for 20 min, prehybridized at 42°C for 1 h, and hybridized with DIG-labeled riboprobes (500 μg/ml) at 58°C overnight. After hybridization, the sections were washed and blocked with blocking reagent (Roche Diagnostics). DIG was detected with an alkaline phosphatase-conjugated anti-DIG antibody (Roche Diagnostics; diluted 1:1,000) and chromogenic development was conducted with NBT/BCIP (Roche Diagnostics). The samples were dehydrated by a series of graded ethanol, cleared in xylene, sealed with neutral resin and taken with a microscope (Olympus, Japan).

### Physiological Functions

#### Fasting Challenge

Approximately 40 basses per group (for short term fasting challenge, 100.00 ± 12.32 g) and 120 fish per group (for long term fasting challenge, 5.88 ± 0.26 g) were acclimatized in the aquaria at 25.2°C for at least 1 week. In short-term fasting, six basses per group (three duplicated groups were set) were sampled at 0, 1, 6, 12, and 24 h post fasting. In long-term fasting, 120 individuals of spotted sea bass were randomly divided into six 120-l-rectangular containers. Three to six fish per container were sampled after 8-week's fasting. All sampled fish were treated with eugenol and sampled immediately. Brain tissues were collected and temporarily placed into liquid nitrogen and finally stored at −80°C for mRNA extraction.

#### *In vitro* Studies of the MC4R-Regulated Gene Expression

Spotted sea basses were anesthetized with MS-222 before decapitation. Brain tissue was removed and washed three times with phosphate buffered saline (PBS). Brain tissue was separated into small pieces in the tubes containing 500 μl trypsin (0.25%, Biological Industries, Kibbutz Beit Haemek, Israel) and then digested 5 min with 5 ml trypsin. After centrifugation for 5 min at 1,500 rpm, the supernatant was discarded and 50 mL medium containing 10 mL fetal bovine serum (FBS, Biological Industries), 500 μl antibiotics (Biological Industries) containing 100 U/mL penicillin and 100 mg/mL streptomycin, 39.5 mL M199 (Biosharp, Beijing, China) was added into each tube. The homogeneous cell suspensions were filtered into tubes by the screen mesh (40 μm Nylon, Corning, NY, USA). Finally, 1 mL cell suspension was added to each well of the 24-well plate and cells were incubated at 28°C. After 36 h incubation, α-MSH was added with fresh media with 20% FBS. Brain cells were collected after incubation and stored at −80°C for RNA extraction and qRT-PCR.

### Pharmacological Characterization

#### Ligands, Cell Culture, and Transfection

NDP-MSH, α-MSH, ACTH (1-24), and THIQ were used. NDP-MSH was purchased from Peptides International (Louisville, KY, USA), α-MSH was purchased from Pi Proteomics (Huntsville, AL, USA), ACTH (1-24) from Phoenix Pharmaceuticals (Burlingame, CA, USA), THIQ was purchased from Tocris Bioscience (Ellisville, MO, USA). ^125^I-NDP-MSH was iodinated as previously described (34) and ^125^I-cAMP was iodinated in our lab via chloramine T method (35, 36).

The N-terminal c-myc-tagged wild-type hMC4R subcloned into pcDNA3.1 was described previously (37). Open reading frame (ORF) of *Lm*MC4R was identified by transcriptome data and the N-terminal c-myc-tagged ORF of *Lm*MC4R was then subcloned into pcDNA3.1 vector by GenScript (Piscataway, NJ, USA) to obtain the plasmid expressing *Lm*MC4R.

Three peptide ligands (α-MSH, ACTH (1-24) and NDP-MSH) and one small molecule ligand (THIQ) were used to evaluate ligand binding and signaling properties of *Lm*MC4R. α-MSH and ACTH are endogenous agonists of all MCRs with the exception that α-MSH cannot activate MC2R. In this study, ACTH (1-24) instead of full-length ACTH (1-39) was used to investigate the pharmacological characteristics of *Lm*MC4R as the first 24 amino acids of ACTH are highly conserved in different species ranging from human to teleosts and ACTH (1-24) is equipotent to the full-length ACTH (38, 39). THIQ is small molecule ligand of hMC4R and our previous studies revealed that they act as pharmacological chaperones, rescuing intracellularly retained hMC4R mutants (7, 40).

Human Embryonic Kidney (HEK) 293T cells (Manassas, VA, USA) were cultured and used for pharmacological assays. Plasmid transfection was performed as described before (41). At 48 h after transfection, binding, and signaling assays were performed.

#### Cell Surface and Total Expressions of hMC4R and LmMC4R

HEK293T cells were transiently transfected with hMC4R or *Lm*MC4R plasmid with N-terminal c-myc tag. Forty-eight hours after transfection, cells were incubated with mouse anti-myc 9E10 monoclonal antibody (Developmental Studies Hybridoma Bank, The University of Iowa, Iowa City, IA, USA) diluted 1:40 for 1 h. Cells were then washed and incubated with Alexa Fluor 488-labeled goat anti-mouse antibody (Invitrogen, Grand Island, NY, USA) diluted 1:2,000 for 1 h. The C6 Accuri Cytometer (Accuri Cytometers, Ann Arbor, MI, USA) was used for analysis. Fluorescence of cells expressing the empty vector (pcDNA3.1) was used for background staining. The expression of the *Lm*MC4R was calculated as percentage of hMC4R expression using the following formula: [(*Lm*MC4R – pcDNA3.1)/(hMC4R – pcDNA3.1) ×100%] (42).

#### Ligand Binding Assays

For ligand binding, 48 h after transfection, cells were washed twice with warm DMEM containing 1 mg/mL bovine serum albumin (referred herein as DMEM/BSA). Subsequently, DMEM/BSA without or with different concentrations of unlabeled ligands and 80,000 cpm of [^125^I]-NDP-MSH were added to each well, with the total volume of 1 ml, and then the cells were incubated at 37°C for 1 h. The final concentration of various unlabeled ligands ranged from 10^−11^ to 10^−6^ M for NDP-MSH and THIQ, or from 10^−10^ to 10^−5^ M for α-MSH and ACTH (1-24). After incubation, the cells were washed twice with cold Hank's balanced salt solution containing 1 mg/mL BSA to terminate the reaction. Cells were then lysed by 0.5 M NaOH and collected for radioactive assays by gamma counter (Cobra II Auto-Gamma, Packard Bioscience, Frankfurt, Germany).

#### Ligand Stimulated cAMP Production

For intracellular cAMP evaluation, 48 h after transfection, HEK293T cells were washed twice with warm DMEM/BSA and then incubated with warm DMEM/BSA containing 0.5 mM isobutylmethylxanthine (Sigma-Aldrich) for 15 min. Subsequently, different concentrations of ligands were added to each well, with the total volume of 1 ml, to evaluate the ligand-stimulated intracellular cAMP levels. Final concentration of various unlabeled ligands ranged from 10^−12^ to 10^−6^ M. After 1 h incubation, the reaction was terminated on the ice and intracellular cAMP was collected by adding 0.5 M percholoric acid containing 180 μg/ml theophylline (Sigma-Aldrich) and 0.72 M KOH/0.6 M KHCO_3_ into each well. Intracellular cAMP levels were determined by radioimmunoassay as previously described (35) and ^125^I-cAMP was iodinated via chloramine T method (35, 36).

### Statistical Analysis

SPSS19.0 software was used to calculate the mean and standard error of the mean (S.E.M.) of gene expression results and results are presented as mean ± S.E.M. Significant differences of gene expression were determined by one-way ANOVA followed by Duncan's multiple range test with the significance level set at *P* < 0.05. GraphPad Prism 4.0 software (San Diego, CA, USA) was used to calculate the ligand binding and cAMP signaling parameters such as maximal binding (B_max_) and IC_50_ of ligand binding and maximal response (R_max_) and EC_50_ of cAMP signaling. The significance of differences in B_max_, IC_50_, R_max_ and EC_50_ between *Lm*MC4R and hMC4R were determined by Student's *t*-test by GraphPad Prism 4.0 software.

## Results

### Nucleotide and Deduced Amino Acid Sequences of *Lm*MC4R

**Figure 1:**
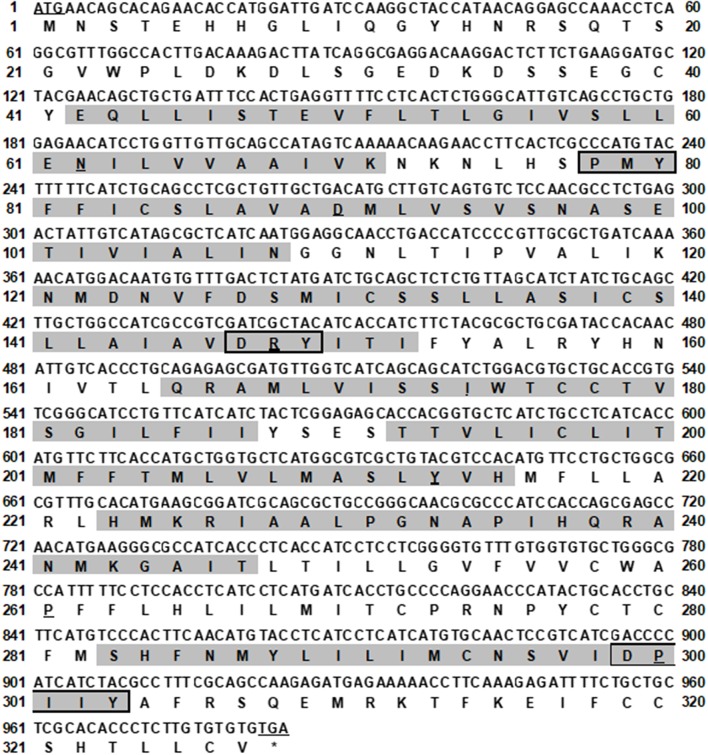
Nucleotide and deduced amino acid sequence of *Lm*MC4R. Positions of nucleotide and amino acid sequences are indicated on both sides. The seven TMDs are shaded in gray. The conserved motifs (PMY, DRY, and DPIIY) are highlighted in boxes. Underlines show initiation codon and most conserved residues in each TMD. Asterisk (*) denotes stop codon.

**Figure 2:**
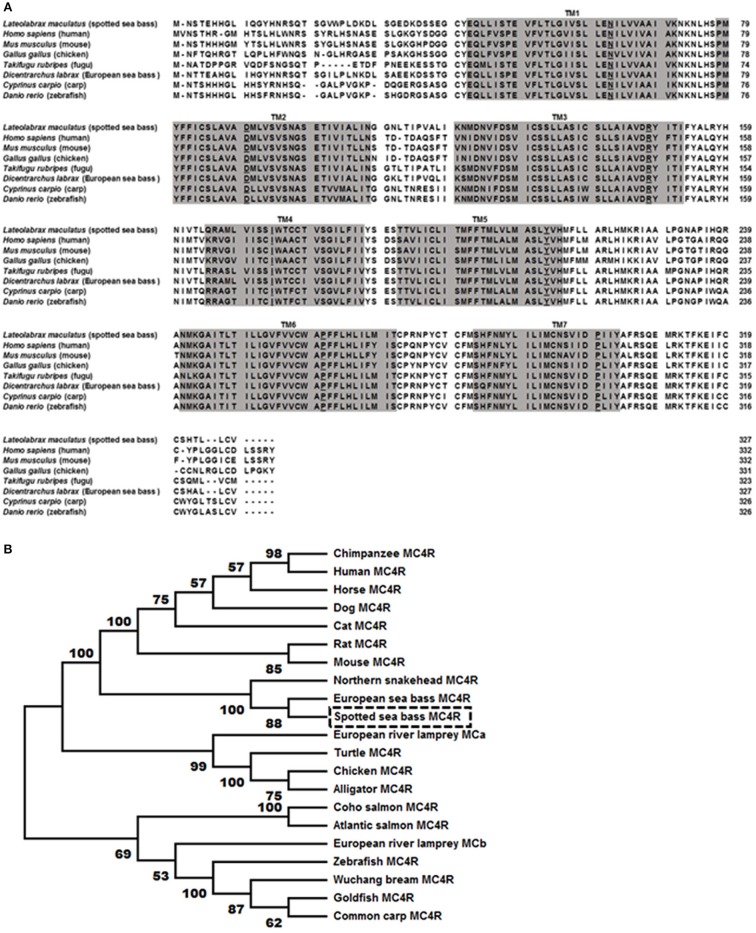
Comparison of amino acid sequences between *Lm*MC4R and MC4Rs from other species **(A)** and phylogenetic tree of MC4R proteins **(B)**. In **(A)**, amino acids in shaded boxes indicate putative TMD 1-7, the most conserved residues in each TMD are underlined. In **(B)**, trees were constructed using the neighbor-joining (NJ) method. Box shows *Lm*MC4R. GenBank accession numbers: alligator (XP_006025279.1); Atlantic salmon (XP_014036044.1); cat (BBD19891.1); chicken (NP_001026685.1); chimpanzee (PNI69802.1); coho salmon (XP_020349696.1); common carp (CBX89936.1); dog (NP_001074193.1); European river lamprey MCRa (ABB36647.1); European river lamprey MCRb (ABB36648.1); European seabass (CBN82190.1); goldfish (CAD58853.1); horse (XP_001489706.1); human (NP_005903.2); mouse (NP_058673.2); Northern snakehead (AMM02541.1); rat (NP_037231.1); turtle (XP_024059247.1); Wuchang bream (AWA81516.1); zebrafish (NP_775385.1).

The bass *mc4r* gene sequence was identified from transcriptome databases (GenBank: SRR4409341/SRR4409397) (43). The total cDNA sequence of bass *mc4r* was 1,588 bp, containing an ORF of 984 bp that encoded a putative protein of 327 amino acids. We identified that the 5′ and 3′ untranslated region of bass *mc4r* was 493 and 114 bp, respectively. Like other GPCRs, *Lm*MC4R had seven putative hydrophobic transmembrane domains (TMDs) with three extracellular loops (ECLs) and three intracellular loops (ICLs, Figure 1). The deduced amino acid sequence of TMDs, ECLs, and ICLs were significantly conserved to those of other species. The predicted amino acid sequence of *Lm*MC4R was 94, 88, 83, 82, 70, 70, and 70% identical to European sea bass (*Dicentrarchus labrax*), fugu (*Takifugu rubripes*), common carp (*Cyprinus carpio*), zebrafish (*Danio rerio*), human (*Homo sapiens*), chicken (*Gallus gallus*) and mouse (*Mus musculus*) MC4Rs, respectively (Figure 2A). Phylogenetic tree analysis between *Lm*MC4R and MC4Rs in other vertebrates revealed that *Lm*MC4R was localized in a clade of teleost MC4Rs and was evolutionarily closer to European sea bass MC4R (Figure 2B).

### Bass *mc4r* mRNA Tissue Distribution and Localization in Brain

**Figure 3:**
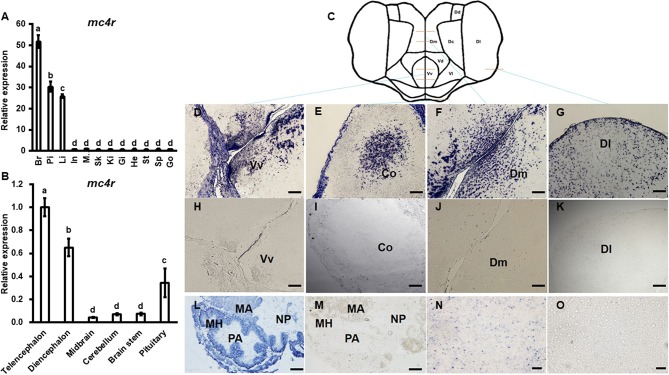
Expression of bass *mc4r* mRNA in various tissues **(A)** and brain regions **(B)** and localization in brain **(D–K)**, pituitary **(L,M)** and liver **(N,O)**. Data are presented as means ± S.E., *n* = 6. Different letters indicate significant difference (*P* < 0.05, one-way ANOVA followed by Duncan's multiple range test). In, intestines; Pi, pituitary; Mu, muscle; Br, brain; Sk, skin; Sp, spleen; Li, liver; Gi, gill; Ki, kidney; St, stomach; He, heart; Go, gonad. The structure and cut regions of telencephalon is shown in **(C)**. **(D–O)** show the bass *mc4r* mRNA localization in brain, pituitary and liver, respectively. Hybridization of sense bass *mc4r*-cRNA probe did not display specific signals in the brain samples **(H–K,M,O)**, showing specificity of the signal. Vv, ventral telencephalon; Co, central region of the olfactory bundle; DI, lateral part of the dorsal telencephalon area; Dm, medial part of the dorsal telencephalon part. PA, pro-adenohypophysis; MH, meso-adeno hypophysis; MA, meta adenohypophysis; NP, neurohypophysis. Scale bars of figs. **(D–K)** indicate 50 μm and figs. **(L–O)** indicate 20 μm.

Bass *mc4r* mRNA expression in brain and peripheral tissues (pituitary, intestine, muscle, skin, spleen, liver, gill, kidney, stomach, heart and gonad) was analyzed by qRT-PCR (Figure 3A). The expression of *18s* mRNA, a stable reference gene, was used as an internal control for normalization. Bass *mc4r* mRNA was highly expressed in the brain, followed by pituitary, liver and other peripheral tissues.

The relative mRNA expression of bass *mc4r* was evaluated in different brain regions (Figure 3B). Higher *mc4r* mRNA expression was observed in telencephalon, diencephalon and pituitary gland (Figure 3B). *In situ* hybridization showed that *mc4r* was localized in the ventral part of the ventral telencephalon (Vv) (Figures 3D,H). The *mc4r-*expressing cells were observed in the brain regions of the central region of the olfactory bundle (Co) (Figures 3E,I), the lateral part of the dorsal telencephalon area (Dl) (Figures 3G,K), and the medial part of the dorsal telencephalon part (Dm) (Figures 3F,J). We also observed the localization of bass *mc4r* mRNA in cells of pituitary (Figures 3L,M) and liver (Figures 3N,O).

### Change in Bass *mc4r* mRNA Expression in Fasting Challenge

**Figure 4:**
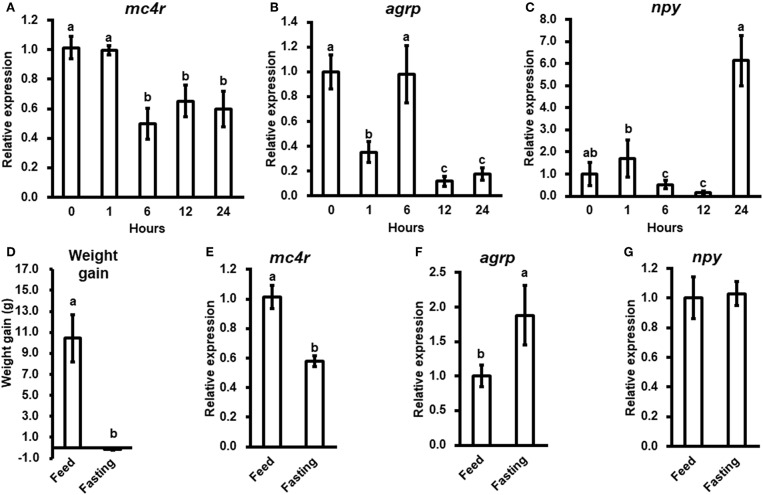
The relative mRNA expression of brain *mc4r, agrp* and *npy* during short-term **(A–C)** and long-term **(E–G)** fasting challenge and body weight of spotted sea bass in long-term fasting challenge **(D)**. Data are presented as means ± S.E., *n* = 6. Different letters indicate significant difference (*P* < 0.05, one-way ANOVA followed by Duncan's multiple range test).

To evaluate the potential physiological functions of bass *mc4r* in regulating energy homeostasis, we analyzed brain *mc4r* mRNA expressions in short-term and long-term fasting experiments. Short-term fasting did not change body weight (data not shown). Although there was no change at 1 h after fasting, there was significant decrease in *mc4r* expression at 6, 12, and 24 h (Figure 4A). Expression of *agrp* was significantly decreased at 1, 12, and 24 h (Figure 4B), and expression of *npy* was significantly decreased at 6 and 12 h (Figure 4C).

In long-term fasting experiment, when compared to the initial body weight (5.88 ± 0.26 g), spotted sea bass in feeding group increased body weight (16.32 ± 2.23 g) by 10.44 g, while spotted sea bass in fasting group decreased body weight (5.73 ± 0.07 g) by 0.15 g (Figure 4D). We observed that *mc4r* expression was significantly down-regulated (Figure 4E), *agrp* mRNA expression was significantly up-regulated (Figure 4F) while there was no significant change in *npy* expression (Figure 4G).

### *In vitro* Studies of the *Lm*MC4R Regulated Gene Expression

**Figure 5:**
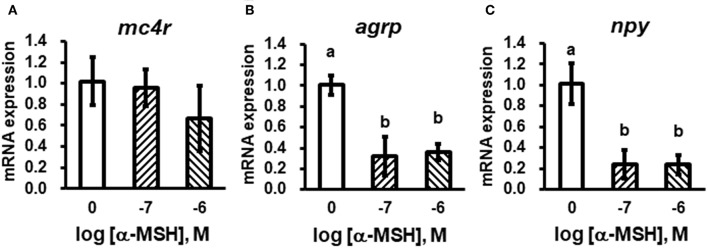
*In vitro* evaluation of bass *mc4r* **(A)**, *agrp* **(B)**, and *npy* **(C)** mRNA expression in isolated brain cells. Data are presented as means ± S.E., *n* = 6. Different letters indicate significant differences compared to control (*P* < 0.05, student *t*-test).

We stimulated isolated brain cells (Figure S1) with 10^−7^ or 10^−6^ M α-MSH for 3 h. We showed that *mc4r* mRNA expression was not altered (Figure 5A), while mRNA expression of *agrp* and *npy* was significantly down-regulated (Figures 5B,C).

### Cell Surface Expression and Ligand Binding Properties of *Lm*MC4R

**Figure 6:**
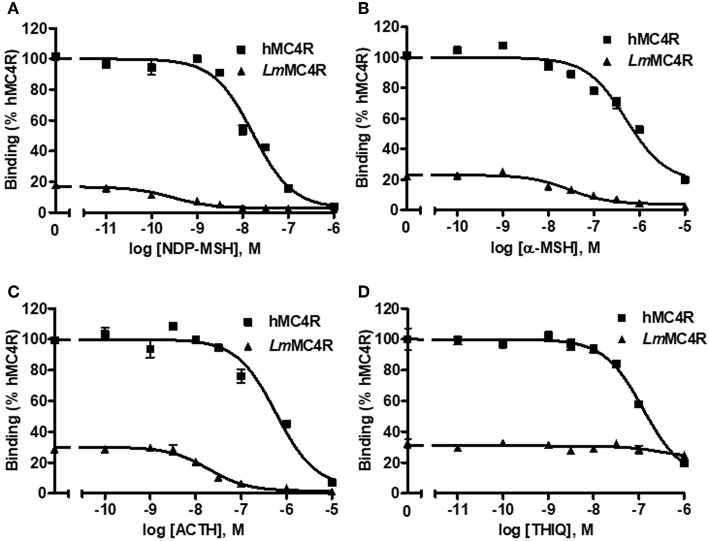
Ligand binding properties of *Lm*MC4R. HEK293T cells were transiently transfected with hMC4R or *Lm*MC4R plasmids. Forty-eight hours after transfection, different concentrations of unlabeled NDP-MSH **(A)**, α-MSH **(B)**, ACTH (1-24) **(C)**, and THIQ **(D)** were used to displace the binding of ^125^I-NDP-MSH, rescpectively. Data are expressed as % of hMC4R binding ± range from duplicate measurements within one experiment. The curves are representative of 3 independent experiments.

The cell surface and total expressions of *Lm*MC4R were only ~2.1% to those of hMC4R, showing significant difference (Figure S2). Competitive ligand binding assays were performed to investigate the binding property of *Lm*MC4R. Different concentrations of unlabeled ligands including α-MSH, ACTH (1-24), NDP-MSH, and THIQ, were used to compete with a fixed amount of ^125^I-NDP-MSH. Maximal binding value of the *Lm*MC4R was around 20% of that of the hMC4R (Figure 6 and Table 2). Both hMC4R and *Lm*MC4R bound to NDP-MSH with the highest affinity. When unlabeled α-MSH, NDP-MSH or ACTH (1-24) was used as the ligand, *Lm*MC4R showed significantly lower IC_50_ values compared to those of hMC4R (Table 2). When THIQ was used as the unlabeled competitor, *Lm*MC4R was not able to displace ^125^I-NDP-MSH, whereas dose-dependent displacement of ^125^I-NDP-MSH binding to the hMC4R was observed (Figure 6D and Table 2).

**Table 2:** The ligand binding properties of *Lm*MC4R.

	**B_**max**_ (%)**	**NDP-MSH**	**α-MSH**	**ACTH**	**THIQ**
		**IC_**50**_ (nM)**	**IC_**50**_ (nM)**	**IC_**50**_ (nM)**	**IC_**50**_ (nM)**
hMC4R	100	18.47 ± 0.85	576.70 ± 0.37	457.83 ± 116.82	156.67 ± 16.04
*Lm*MC4R	22.66	0.31 ± 0.03^b^	31.95 ± 4.86^b^	24.66 ± 5.55^a^	N/A^c^

a *Significantly different from the parameter of hMC4R, P < 0.05*.

b *Significantly different from the parameter of hMC4R, P < 0.001*.

c *Could not be determined*.

### Signaling Properties of *Lm*MC4R

**Figure 7:**
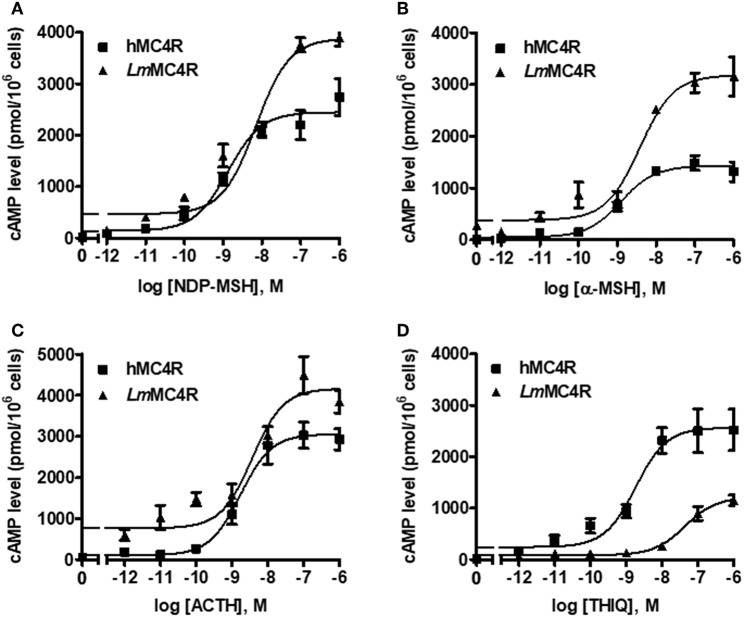
Signaling properties of *Lm*MC4R. HEK293T cells were transiently transfected with hMC4R or *Lm*MC4R plasmids. Forty-eight hours after transfection, different concentrations of NDP-MSH **(A)**, α-MSH **(B)**, ACTH (1-24) **(C)**, and THIQ **(D)** was used to stimulate the cells transfected with hMC4R or *Lm*MC4R. Data are expressed as mean ± SEM from triplicate measurements within one experiment. The curves are representative of three independent experiments.

Dose-dependent increase of intracellular cAMP was observed when *Lm*MC4R was stimulated by NDP-MSH, α-MSH, ACTH (1-24) and THIQ (Figure 7). The maximal responses of *Lm*MC4R in response to NDP-MSH, α-MSH, and ACTH were 144.57 ± 8.65%, 216.86 ± 16.88%, and 125.56 ± 5.45%, respectively, of those of hMC4R, whereas the maximal response of *Lm*MC4R in response to THIQ was 57.78 ± 5.32% of that of hMC4R (Figure 7 and Table 3). EC_50_s of NDP-MSH and THIQ for *Lm*MC4R were significantly higher than those for hMC4R, and EC_50_s of α-MSH and ACTH (1-24) for *Lm*MC4R and hMC4R were not significantly different (Figure 7 and Table 3).

**Table 3:** The cAMP signaling properties of *Lm*MC4R.

	**Basal (%)**	**NDP-MSH**		**α-MSH**		**ACTH**		**THIQ**	
		**EC_**50**_ (nM)**	**R_**max**_ (%)**	**EC_**50**_ (nM)**	**R_**max**_ (%)**	**EC_**50**_ (nM)**	**R_**max**_ (%)**	**EC_**50**_ (nM)**	**R_**max**_ (%)**
hMC4R	100	1.22 ± 0.08	100	2.69 ± 0.37	100	2.13 ± 0.49	100	2.31 ± 0.24	100
*Lm*MC4R	891 ± 251	5.09 ± 1.06^a^	144.57 ± 8.65	5.20 ± 0.80	216.86 ± 16.88	2.72 ± 0.51	125.56 ± 5.45	63.88 ± 11.24^b^	57.78 ± 5.32

a *Significantly different from the parameter of hMC4R, P < 0.05*.

b *Significantly different from the parameter of hMC4R, P < 0.01*.

## Discussion

In this study, we demonstrated that spotted sea bass *mc4r* encoded a protein of 327 amino acids with seven transmembrane domains and conversed motifs such as PMY, DRY, and DPIIY (DPxxY) (Figure 1). Compared to transmembrane domains, extracellular N-terminal domain and ECLs were less conservative (Figure 2). Similar results have also been reported in other teleosts (20–22, 44, 45). Cys residues have been shown to be critical for MC4R integrity possibly by forming disulfide bonds (46). We identified 15 Cys residues in *Lm*MC4R, as in other teleost MC4Rs (19, 21, 45), suggesting that the number of Cys residues was highly conserved in MC4Rs during teleost evolution. Amino acid sequence of *Lm*MC4R was ~94% identical to European sea bass MC4R, ~80% identical to several other teleost MC4Rs including zebrafish, fugu and carp, and was ~70% identical to mammalian MC4Rs (Figure 2).

We observed the highest *mc4r* expression in brain (Figure 3). This is consistent with previous studies that non-mammalian *MC4Rs* are also abundantly expressed in brain (36). Expression patterns of the teleost *mc4r* are much wider when compared to those in mammals. In addition to brain, teleost *mc4r* are also expressed in pituitary and certain peripheral tissues including eyes, liver, gonads, spleen, and gastrointestinal tract (36). The spotted sea bass *mc4r* was also highly expressed in pituitary, similar to findings in barfin flounder, goldfish, zebrafish, and European sea bass (36). Recent studies showed teleost MC4Rs might play important role in regulating gonadal development (21, 22). In this study, we observed that *mc4r* expression in spotted sea bass gonad was low (Figure 3). Taken together, these results suggested that wider expression of teleost *mc4r* might be associated with roles in regulating multiple physiological functions.

In mice, changes in food intake represent 60% of the total effect of the MC4R in regulating energy homeostasis (47). In Mexican cavefish (*Astyanax mexicanus*), *mc4r* mutations associated with signaling efficiency contribute to physiological adaptations to nutrient-poor conditions by increasing appetite, growth, and starvation resistance (24). We observed short-term fasting led to down-regulation of *mc4r* with fluctuating changes in *agrp* and *npy* expression while long-term fasting resulted in down regulated *mc4r* with up-regulated *agrp* (Figure 4). We hypothesize that MC4R might be more important in regulating long-term energy balance. Moreover, *in vitro* studies showed incubation of isolated brain cells with α-MSH could decrease *npy* and *agrp* mRNA expressions, although it did not change *mc4r* expression (Figure 5). All these results showed the conserved function of MC4R in regulating food intake (47). Further studies need to investigate the distinct functions of AGRP or NPY neurons in regulating food intake with MC4R due to the fact they showed different transcriptional patterns during fasting (Figure 4).

Detailed pharmacological studies were further performed on *Lm*MC4R. We observed that the cell surface expression of *Lm*MC4R was significantly lower than that of hMC4R, which might explain the differences of total binding between *Lm*MC4R and hMC4R. Ligand binding experiments also showed that *Lm*MC4R bound to α-MSH and ACTH (1-24) with similar affinities (Figure 6). Compared with hMC4R, *Lm*MC4R showed significantly higher binding affinity to NDP-MSH (~60-fold higher), α-MSH (~20-fold higher), and ACTH (1-24, ~20-fold higher) (Figure 6), consistent with previous studies of swamp eel, spotted scat, orange-spotted grouper, fugu and rainbow trout MC4Rs (21–23, 48, 49).

In cAMP signaling assays, α-MSH and ACTH (1-24) stimulated *Lm*MC4R and hMC4R with similar potencies (Figure 7). THIQ could bind to hMC4R and displace the ^125^I-NDP-MSH in a dose-dependent manner, suggesting that binding sites of THIQ and NDP-MSH were overlapping. THIQ could not displace ^125^I-NDP-MSH binding at *Lm*MC4R; however, THIQ stimulated intracellular cAMP accumulation at *Lm*MC4R with an EC_50_ of 63.88 nM, which was significantly higher than that of hMC4R. We propose that THIQ might act as an allosteric agonist at *Lm*MC4R, similar to our previous studies in grass carp and swamp eel (21, 22).

In agreement with previous studies that teleost MC4Rs showed high constitutive activity in cAMP pathway (20–23), this study observed that *Lm*MC4R had ~9-fold higher constitutive activity than that of hMC4R (Figure 7 and Table 3). N-termini act as an important modulator in regulating constitutive activities in GPCRs (50–52). Although amino acid sequences of MC4Rs are conserved from mammals to teleosts, N-termini of *Lm*MC4R and other teleost MC4Rs were less conserved to those of hMC4R, raising the possibility that variations of residues in N-termini might lead to high constitutive activities in teleost. Indeed, hMC4R has also been shown to have constitutive activity (53) and mutations leading to decreased constitutive activity or other loss-of-functions are believed to be associated with obesity pathogenesis (16, 54, 55). However, in agriculture (aquaculture), the farmed animals with lower MC4R constitutive activity may show a higher food efficiency, lower basal metabolism and faster weight gain, increasing the economic benefits of agriculture (aquaculture). Inverse agonists that decrease fish MC4R constitutive activity might be used in aquaculture.

## Data Availability Statement

The raw data supporting the conclusions of this manuscript will be made available by the authors, without undue reservation, to any qualified researcher.

## Author Contributions

YL, Y-XT, H-SW, and XQ: conceptualization. K-QZ, Z-SH, and W-JL: project administration. YL and Y-XT: supervision. YL, Y-XT, and XQ: methodology. Z-SH and K-QZ: writing—original draft. YL, Y-XT, H-SW, and XQ: writing—review and editing.

### Conflict of Interest

The authors declare that the research was conducted in the absence of any commercial or financial relationships that could be construed as a potential conflict of interest.

## Abbreviations

ACTH: adrenocorticotropic hormone
AgRP: Agouti-related peptide
ECL: extracellular loop
FBS: fetal bovine serum
GH: growth hormone
GHRH: growth hormone-releasing hormone
GPCR: G protein-coupled receptor
HEK 293T cells: human embryonic kidney 293T cells
hMC4R: human MC4R
ICL: intracellular loop
i.c.v.: intracerebroventricular
IGF: insulin-like growth factor
MCR: melanocortin receptor
MSH: melanocyte-stimulating hormone
NDP-MSH, [Nle^4^: D-Phe^7^]-α-MSH
NPY: neuropeptide-Y
ORF: open reading frame
PBS: phosphate buffered saline
PKA: protein kinase A
SST: somatostatins
THIQ, N-[(3R)-1, 2, 3, 4-tetrahydroisoquinolinium-3-ylcarbonyl]-(1R)-1-(4-chlorobenzyl)-2-[4-cyclohexyl-4-(1H-1, 2: 4-triazol-1-ylmethyl)piperidin-1-yl]-2-oxoethylamine
TMD: transmembrane domain.
