# Supplementary material for: Melanocortin-4 Receptor in Spotted Sea Bass, Lateolabrax maculatus: Cloning, Tissue Distribution, Physiology, and Pharmacology
Source: Front Endocrinol (Lausanne). 2019 Oct 18;10:705. doi: 10.3389/fendo.2019.00705 (PMC6813543; doi:10.3389/fendo.2019.00705)
Supplement: Supplementary file 1 [file Data_Sheet_1.docx]

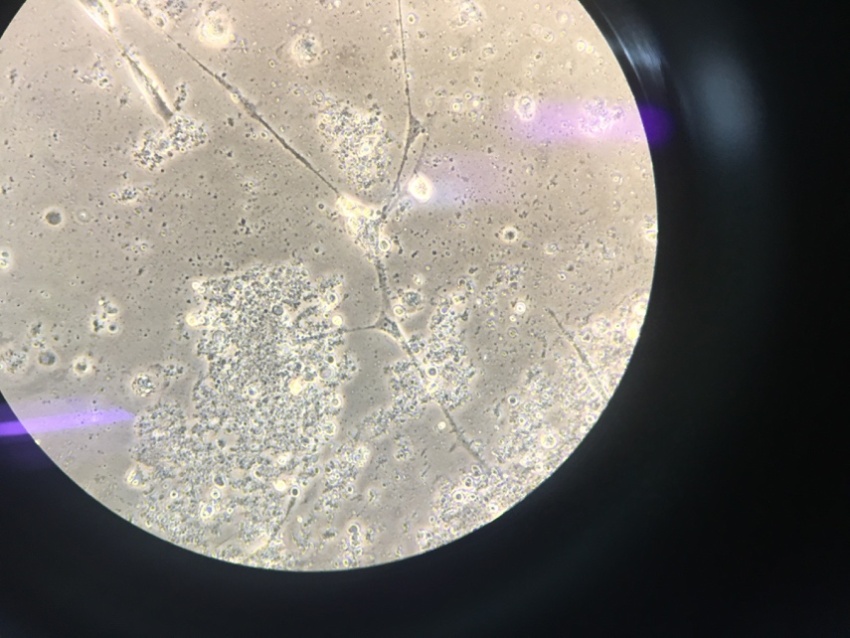


**Fig. S1. Spotted sea bass brain cells. Arrow indicates neural cell (black) and nerve fiber (purple).**

**
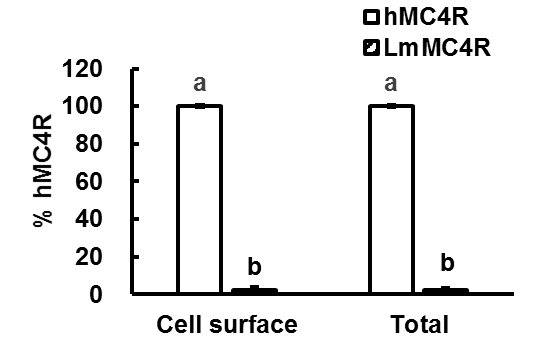
**

**Fig. S2. Cell surface and total expression of *Lm*MC4R.** HEK293T cells were transiently transfected with hMC4R or *Lm*MC4R plasmids. Forty-eight hours after transfection, cells are stained with Alexa Fluor 488-conjugated antibody. The immunostaining was measured using a C6 Accuri Cytometer. The results are expressed as % of hMC4R cell surface/total expression level after correction of the non-specific staining in cells expressing the empty vector. Results are shown as mean ± SEM of at least three independent experiments. Different letters indicate significant differences compared to hMC4R (*P* < 0.05, Student’s t test).
